# Supplementary material for: The Dct−/− Mouse Model to Unravel Retinogenesis Misregulation in Patients with Albinism
Source: Genes (Basel). 2022 Jun 27;13(7):1164. doi: 10.3390/genes13071164 (PMC9324463; doi:10.3390/genes13071164)
Supplement: Supplementary file 1 [file genes-13-01164-s001.zip › genes-1764580 supplementary.pdf]

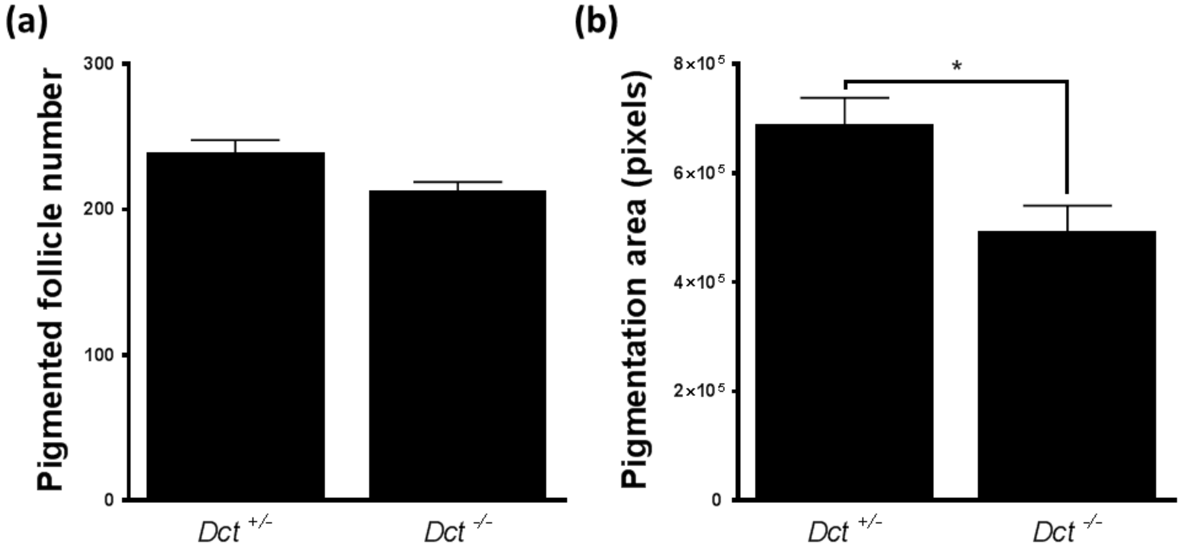

Figure S1. Pigmented hair follicle analysis at P2. Quantification was performed on images, as shown in Figure 2c, taken from $n = 3$ animals for each genotype. Quantitative analysis was performed with ImageJ. All images were converted to 8-bit grayscale format and thresholded after subtraction of fixed background level. The "Analyze particles" command was used to generate quantitative values. The statistical significance of the difference in mean values was determined by Student's t-test. (**a**) At P2, there was no significant difference in the number of pigmented hair follicles in *Dct* -/- mice compared to control littermates; (**b**) Pigmentation coverage was found significantly lower in *Dct*-/- hair follicles, * indicates $p < 0.05$.

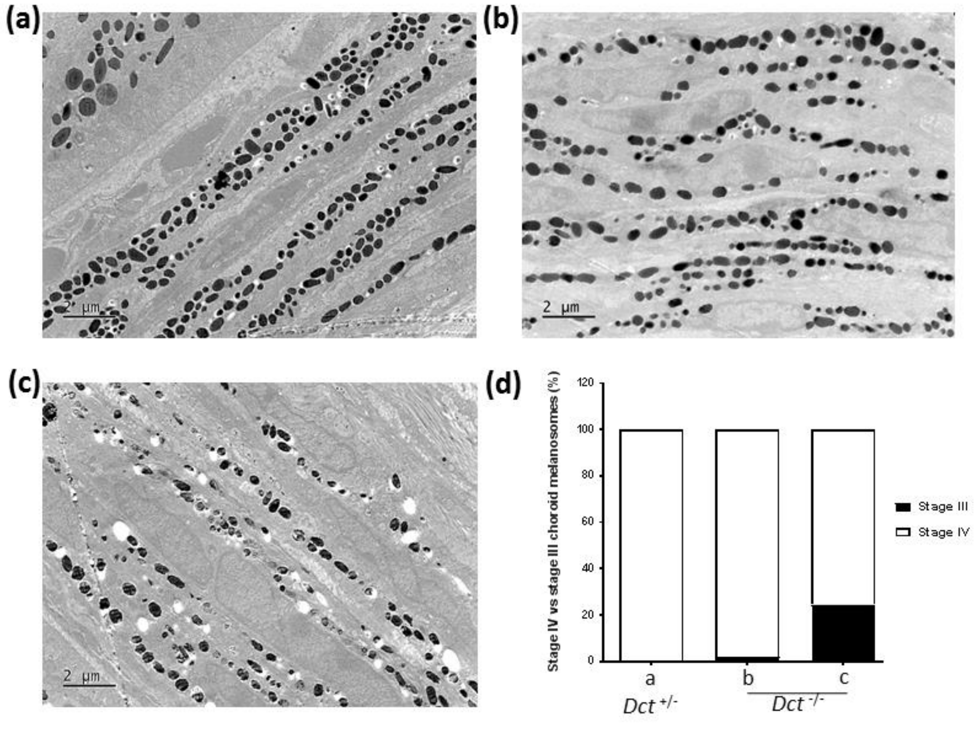

Figure S2. Transmission electron microscopy of the choroid at 1 month. (**a**) *Dct*$^{+/-}$, the choroid is homogeneously pigmented with stage IV melanosomes; (**b**) The choroid of *Dct*$^{-/-}$ mice displayed a normal ultrastructure except in a few fields like in (**c**), where melanosomes were less dense and/or less mature. (**d**).Quantification of stage IV versus stage III melanosomes in the 3 images.

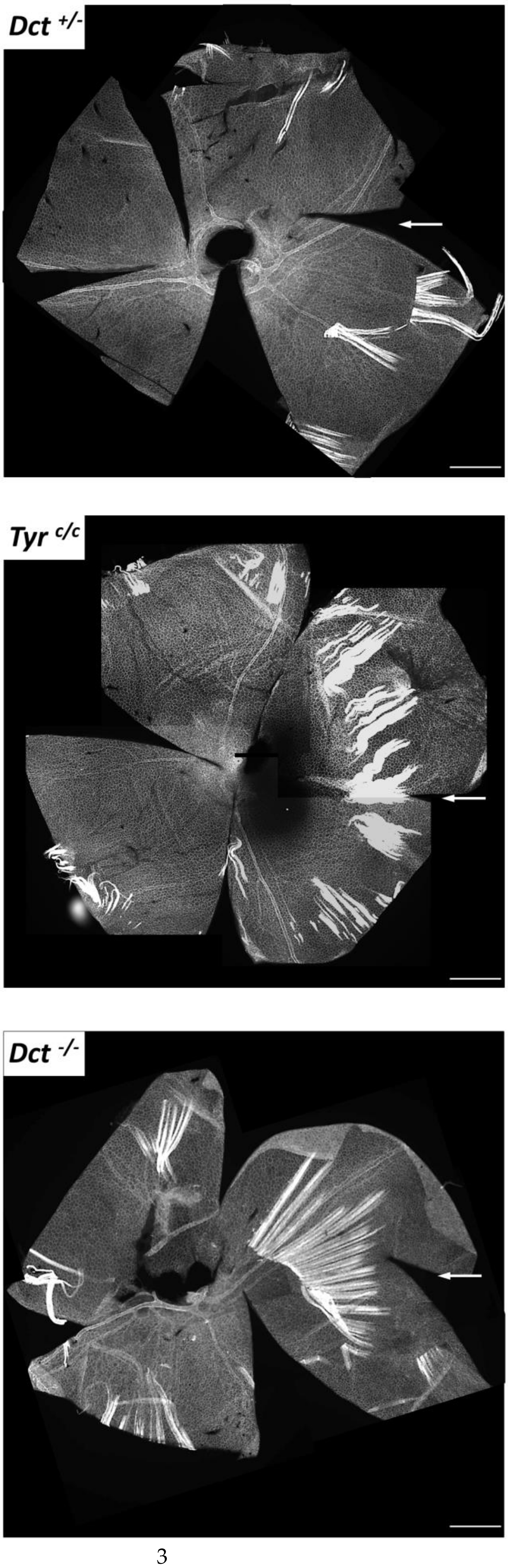
Dct +/-
Tyr c/c
Dct -/-

Figure S3. Whole RPE flat mounts of P0.5 mice after labeling with Alexafluor 488 phalloidin. As much extraocular tissue as possible was removed, which was more difficult to achieve on non-pigmented eyes so that most of the surface could be used for cell size estimation. The white arrow indicates the nasal pole.
